# Research on policy effect and regional difference of allocation efficiency of rural preschool education resources: evidence from China

**DOI:** 10.1371/journal.pone.0301064

**Published:** 2025-05-19

**Authors:** Miao miao Tang, Qiang Lan

**Affiliations:** 1 School of Educational Science, Northwest Normal University, Lanzhou, China; 2 School of Future Teacher, Guangxi Science & Technology Normal University, Laibin, China; Huanggang Normal University, CHINA

## Abstract

By the end of 2020, with all rural residents living in poverty under the current standard lifted out of absolute poverty, marking a new phase in China’s anti-poverty efforts with new pursuit of the consolidation and expansion of poverty alleviation achievements in the effective connection with rural revitalization. The development and improvement of funding mechanisms for rural preschool education are crucially important to further promoting rural development. Employing an input-oriented three-stage DEA model and the Malmquist index, this study conducts a static and dynamic analysis of resource allocation performance in rural preschool education across 30 provinces in China (excluding Tibet, Hong Kong, Macao, and Taiwan). The findings reveal that random factors and environmental variables lead to an underestimation of rural preschool education investment performance. Secondly, economically developed regions are not necessarily equipped with higher performance in rural preschool education investment as regional differences stem from the combined effects of various economic, agglomeration, demographic, and scale factors across different areas of the country. Finally, based on these empirical results, this paper proposes policy recommendations to enhance resource allocation performance in China’s rural preschool education.

## 1 Introduction

Since the beginning of the 21st century, interstate competition across economic, educational, scientific, and technological domains has escalated markedly. The foundation of a country’s competitiveness lies in its education and economy. Therefore, scholars have coined the 21st century as the new “double-E” era (i.e., Education and Economy) [1]. Across the globe, governments unanimously recognize the role of rural preschool education in promoting economic development and have formulated diverse educational policies to foster economic growth. For example, Nordic countries have implemented rural preschool education frameworks characterized by high-welfare provisions and educational equity, while neoliberal countries have adopted market-driven approaches to rural preschool education.

Against this backdrop, the Chinese government has steadfastly prioritized the integrated development of education and the economy in alignment with global imperatives. The transition from quantitative expansion to qualitative enhancement, coupled with the pursuit of strategic transformation in rural preschool education, has emerged as a central theme in China’s rural educational development. Social equity is an eternal goal pursued by the people, and educational equity serves as an important cornerstone of social justice. Persistent economic and social disparities, compounded by a rigid urban-rural dual structure, have perpetuated a pronounced polarization in educational resources and quality between urban and rural regions [2]. Constrained by natural conditions and geographical location, rural economies often stagnate, concomitant with acute shortages of educational resources, substandard infrastructure, and inferior teaching quality relative to urban counterparts. This reality positions rural education as the most vulnerable segment of the national educational system.Therefore, the crucial challenge in rural education entails achieving breakthroughs in overall educational standards while concomitantly narrowing the urban-rural educational divide. Preschool education is an essential part of the national education system as it not only fosters individual development but also shapes the nation’s long-term prosperity. In this context, rural preschool education under the implementation of the rural revitalization strategy is particularly significant. The allocation of rural preschool education resources, being a weak link in the development of preschool education, is an important foundation for achieving educational equity. The rational allocation and utilization of rural preschool education resources determines the quality of education, hence exploring the allocation of rural preschool education resources holds significant importance.

The concept of educational resource allocation originates from the core propositions of economics. Currently, there is a consensus in the academic community that “educational resources” refer to the various financial, human, and material resources required for conducting all types of education, while “educational resource allocation” concerns how to distribute these resources reasonably and effectively among different levels and categories of education. Macro-level educational resource allocation is guided by national policy and labor market demands, effectively distributing educational resources to different levels of education to pursue the optimal benefits of educational resource allocation [3]. Micro-level educational resource allocation refers to the optimization of resource utilization within educational institutions under given resource constraints [4]. Observing both macro and micro levels, it is not difficult to see that “efficiency” is always the ultimate goal of educational resource allocation. When total input is held constant, efficiency and output gains can be realized through optimal resource configuration. In Western economics, the “Pareto optimality” is generally employed to measure the efficiency of resource allocation. That is, if an economy is in a state where no one can improve their welfare without sacrificing others’ interests, then this is considered an effective resource allocation or has reached Pareto optimality [5]. Pareto optimality is an ideal state of resource distribution, meaning there is no room for Pareto improvements.

The analysis of investment in rural preschool education resources, as the weakest sector in the development of preschool education, is an important foundation for achieving educational equity. Optimizing the allocation of rural preschool education resources will target at the rationality, fairness, and balance of resource distribution. Its significance lies in narrowing the gap in the inequality of preschool education between rural and urban areas and meeting the high expectations of society members for rural preschool education. Pursuing the value of educational equity and the high-quality development of rural preschool education are among the powerful measures to advance the comprehensive promotion of the rural revitalization strategy. Based on this, this study employs an input-oriented three-stage DEA model and the Malmquist index to conduct static and dynamic analyses of rural preschool education resource investment performance across 30 provinces (excluding Tibet, Hong Kong, Macao, and Taiwan) in China. This scientific and objective assessment of its current resource allocation status yields significant theoretical and practical implications for the high-quality and balanced development of rural preschool education in China.

**Fig 1:**
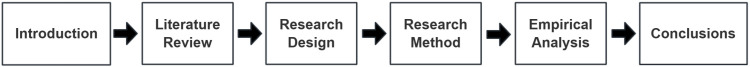
Article structure.

The structure of this paper is shown in Fig 1: Section 1 presents the research background, the research background of this paper. Section 2 provides a comprehensive review of extant literature. Section 3 details the datasets, methodological framework, and indicator systems used in the experiments of this paper. Section 4 elaborates on the research methodology. Section 5 describes the experimental design and presents the results, demonstrating how research questions were addressed. Section 6 concludes by summarizing findings,discussing limitations, and proposing future research directions.

## 2 Literature Review

The contemporary world is at a critical juncture in the development of the knowledge-based economy and globalization. As a primary means to assess and understand the efficiency of educational resource utilization, educational performance evaluation has emerged as a pivotal instrument for refining educational governance models and conducting effective diagnostics. Exploring and interpreting the historical experiences and cutting-edge achievements of advanced countries in educational performance evaluation holds significant implications for improving China’s educational performance evaluation system. The United States has nearly four decades of experience in performance evaluation, traversing three transformative stages: performance assessment, performance reporting, and performance-based funding. Currently, its performance evaluation framework has attained relative stability. Meanwhile, the United Kingdom, one of the world’s most educationally advanced nations, owes its distinction to a well-established and transparent educational quality assurance mechanism. With the continuous evolution of its education assessment system, the UK’s educational quality assurance framework now comprises three key components: internal quality assurance systems, external quality assurance systems, and societal and media oversight. Although Australia’s performance evaluation practices lagged behind those of the UK, due to their shared membership in the Commonwealth and analogous educational systems, Australia’s performance evaluation has inherited British traditions while adapting them to its national context. Japan’s educational performance evaluation is principally designed to facilitate the optimal utilization of teaching and research funds and provide a rationale for securing external financing. Research on international educational performance evaluation offers valuable insights for optimizing China’s educational resource allocation, formulating fiscal policies, enhancing the efficiency and effectiveness of public funding, and establishing novel performance-based funding mechanisms.

The issue of improving the efficiency of educational resource allocation has long been a central concern for scholars worldwide. Concerning the outcomes of theoretical research, scholarly inquiries into educational resource allocation efficiency originated earlier in foreign contexts compared to China. In 1955, Friedman proposed the concept of “education vouchers” to tackle the issue of suboptimal educational quality stemming from government monopoly and bureaucratic inefficiencies in education. He advocated for the introduction of a competitive mechanism in the education sector to enhance institutional dynamism, thereby elevating school management standards and optimizing resource utilization efficiency. In 1962, Friedman critiqued government monopoly in educational resource allocation, arguing that the public education system would result in allocative inefficiencies and resource waste, a perspective that garnered significant scholarly attention [6]. Rodic pioneered the theoretical framework of educational voucher systems, which innovatively introduced a market competition mechanism to foster inter-school competition and enhance educational resource utilization efficiency [7].

Originating from operations research, Data Envelopment Analysis (DEA), which measures the relative effectiveness among multiple decision-making units, was proposed in 1978 by three American operations researchers, Charnes, Cooper, and Rhodes. Since then, scholars worldwide have promoted the study of educational resource allocation efficiency from qualitative analysis to quantitative research. The DEA approach has been widely adopted by scholars globally for assessing educational resource allocation efficiency [8]. Anthanasassou developed an evaluation framework for higher education resource allocation efficiency by incorporating input indicators (e.g., school education costs, student excellence rates, operational conditions, and institutional scale) and output indicators (e.g., graduate counts, postgraduate degrees awarded, and research outcomes). Using DEA, he assessed the resource allocation efficiency of 45 UK universities [9]. In the study of 448 public middle schools in the Netherlands from 2002 to 2007, Brennan developed pedagogical input-output indicators and incorporated environmental variables into the analysis. He decomposed the Malmquist productivity index into technological change, efficiency change, scale efficiency, and environmental factors, revealing that environmental variables significantly influenced productivity outcomes alongside technological, efficiency, and scale factors [10].Tsakiridou administered a questionnaire survey to 17 Swedish primary schools and applied DEA to analyze resource allocation efficiency, finding that just 23% of institutions achieved optimal resource allocation standards [11].

In the realm of research methodologies, scholars worldwide predominantly employ Stochastic Frontier Analysis (SFA) and Data Envelopment Analysis (DEA) to measure efficiency. The former, as a parametric analysis method, derives efficiency estimates through parametric modeling and variable assumptions [12]. While Its advantage lies in effectively evaluating the efficiency of multiple input indicators and a single output indicator, it encounters limitations in measuring efficiency under scenarios of multiple inputs and multiple outputs.The latter, as a non-parametric approach, can measure the relative efficiency of multiple Decision Making Units (DMU) based on statistical data and mathematical programming. The calculation process of the DEA model dispenses with the need for a predefined production function and remains unconstrained by data size, format, or weighting schemes [13], making the measurement more objective. Therefore, it has been widely applied to evaluate the efficiency in the field of rural preschool education. Traditional DEA models construe each DMU as a static entity, overlooking the dynamic variability and interactive relationships among DMUs, thereby impeding accurate efficiency measurement across the entire system. To address the limitations of traditional DEA models, scholars have developed enhanced variants, including the Super-Efficiency DEA model [14], the Slacks-Based Measure (SBM) model [15], the two-stage DEA model [16], and the three-stage DEA model [17]. On this basis, some scholars have also combined the DEA model with the Tobit model to further investigate the determinants of DEA output variables [18]. The aforementioned DEA models are primarily suitable for calculating static efficiency. To meet the requirements of analyzing the dynamic changes of efficiency over time, scholars have introduced the Malmquist index model as a supplement[19].

A synthesis of extant literature reveals that research on rural preschool education resource allocation efficiency can be characterized by three key dimensions: Firstly, scholars attach importance to the relationship between the efficiency of rural preschool education resource allocation and economic development. Based on the context of urbanization, Lauver examined the efficiency of preschool education resource allocation in Pennsylvania. The study found that urbanization has a positive effect on improving the efficiency of preschool education resource allocation and proposed that future focus areas for preschool education resource allocation should be placed in rural counties[20]. Furthermore, the relationship between the efficiency of rural preschool education resource allocation and national policies attracts many scholars. Lzard once studied the government’s monopoly on the allocation of preschool education resources, suggesting that such a monopoly leads to inefficiency and waste of resources [21]. Wu Guangzhi’s empirical analysis identified three critical criteria for evaluating financial system performance: the effectiveness of educational resource allocation, the adequacy of educational services, and the equity of educational resource allocation [22]. Lastly, scholars focus on the relationship between the efficiency of rural preschool education resource allocation and population. Lewin’s empirical investigation assessed the effects of education reform initiatives on low-income populations in African countries through the lens of educational resource distribution, demonstrating the direct impact of government investment in education on anti-poverty policies [23]. Guo Yuanzhi suggested in his research that the allocation of resources should lean forward poor areas and rural schools, thereby addressing the challenge of significant disparities in resource allocation among different regions [24].

In terms of research content, domestic and international scholars can primarily fall into three categories. The first category concerns the efficiency of rural preschool education resource allocation in different countries. Scholars represented by Bhardwaj used the DEA method to explore the efficiency of preschool education resource allocation in India [25]. Husein further analyzed the cost structure issues, effective resource allocation issues, and productivity conditions of different types of kindergartens in the UK [26]. The second category focuses on analyzing resource allocation efficiency within specific urban or economic regions. Akta examined the operational characteristics of preschool institutions in Turkey and Spain, as well as the status of food services and nutrition education, suggesting that cooperation between countries and the transfer of good practices would produce organized, sustainable, and comprehensive nutrition education programs for cultivating healthy children and healthy adults [27]. The third category is the empirical analysis of the efficiency of rural preschool education resource allocation in specific cities or corresponding provinces. Kun Liang and Chunge Lu investigated the supply of public preschool education resources in Guangdong Province, China. They found problems such as insufficient supply of public preschool education resources and a lack of high-quality inclusive private preschool education resources [28]. Jiyan Wang used the population development environment analysis model to predict the demand for educational resources among Beijing’s preschool population from 2020 to 2040. The research results show that during the forecast period, the scale of eligible early childhood education, the number of preschool children in the first three years, the scale of kindergarten construction, and the demand for full-time teachers and caregivers all showed a trend of first increasing and then decreasing [29].

A synthesis of extant literature reveals that research on rural preschool education resource allocation efficiency can be characterized by three key dimensions: First, there is a lack of empirical research related to the policy effects of resource allocation at the preschool education stage in China, with most studies being limited to the theoretical level; Secondly, existing studies often overlook the exclusion of environmental variables. Given the pronounced environmental dependency of preschool education, external factors significantly influence resource allocation efficiency, thereby potentially undermining the validity of efficiency measurements; Third, traditional DEA and its improved models are often used for research, but the static nature of the model itself is ignored, to some extent neglecting the dynamic changes in input and output during specific periods.

Therefore, in order to effectively fill the current research gaps and promote the high-quality and balanced development of global preschool education, this study innovatively proposes to update the existing efficiency evaluation research model to assess the level of rural preschool resource allocation in China and systematically analyze the external factors affecting the efficiency of resource allocation. Leveraging provincial panel data spanning 2012–2020, this research employs an input-oriented three-stage DEA model and the Malmquist index to undertake static and dynamic assessments of rural preschool education resource allocation efficiency in China. It investigates the policy effects of China’s rural preschool education reforms and provides a basis for further deepening the reform of rural preschool education in China and promoting the standardized development of rural preschool education.

## 3 Research Design

Numerous methodologies exist for evaluating rural preschool education allocation efficiency. Guided by a rigorous consideration of statistical data attributes, random errors, and environmental influences, this study employs a three-stage DEA model to enhance efficiency evaluation rigor. This can avoid the impact caused by dimensionality issues to a greater extent, making the efficiency values more in line with reality. By collecting relevant data on rural preschool education, this study analyzes its efficiency level, regional differences, and specific improvement strategies, forming the technical roadmap of this research.

**Fig 2:**
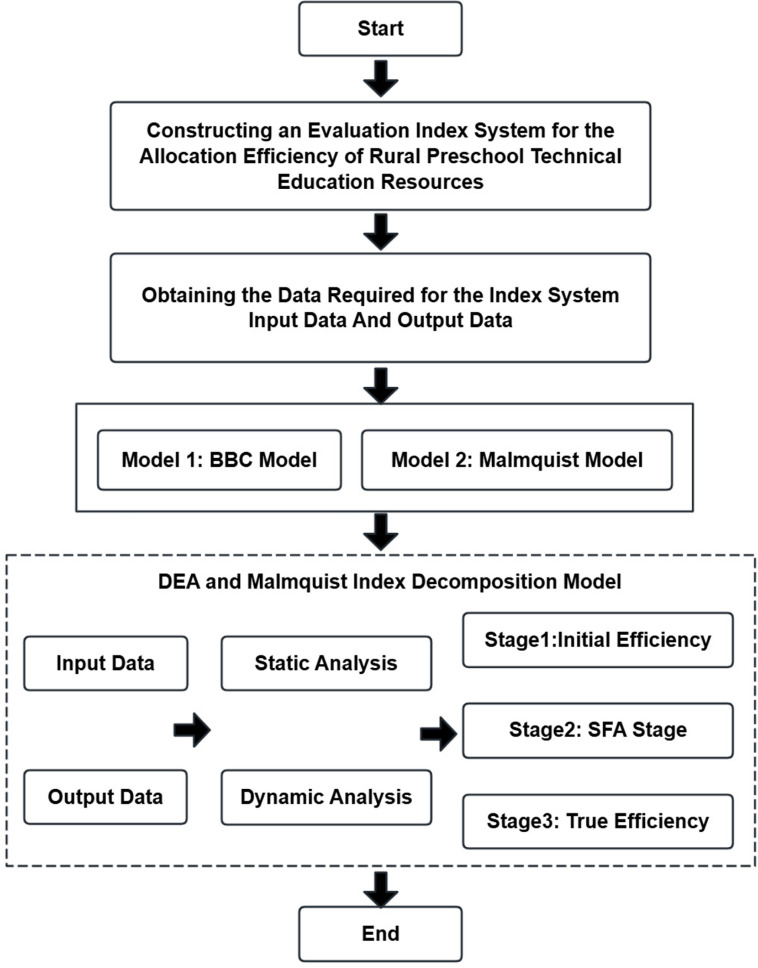
Research technical road map.

The specific ideas proposed in this study include (see Fig 2): 1.Development of an evaluation index system comprising input variables, output variables, and contextual variables for rural preschool resource allocation; 2. Precise measurement of the current efficiency levels of rural preschool education resource allocation; 3. Static analysis of resource allocation efficiency trends and optimal scale configurations; 4.Dynamic assessment of resource allocation efficiency evolution and temporal trends; 5. Investigation into the influences of economic, demographic, agglomeration, and scale factors on rural preschool education resource allocation efficiency.

### 3.1 Index Construction

The DEA model assumes that output monotonically with input. Through the Spearman test by the Stata software, the results show that the test coefficients are all between 0 and 1, indicating a positive correlation between input and output. This finding validates the monotonicity assumption essential for DEA model specification.

On the basis of following the scientific nature and integrity of the data, the indicator system is developed within the framework of “resource input - economic output - external variables”, as shown in Table 1. Since the statistical caliber of the indicators is consistent and not comparable between years, this study conducts deflation adjustments on the indicators to ensure temporal comparability. The input indicators of the rural preschool education resource allocation efficiency encompass per-student education expenditure, the proportion of teachers with tertiary education or higher, per-student school building area, and per-student book holdings; the output indicators include the number of kindergarten graduates and the proportion of people who have received preschool education in the total number of primary school admissions.

**Table 1:** Three stage DEA model index system.

Destination layer	Criteria layer	Variable	Code
The input variables	Financial input	Average education expenditure	Fee
	Human input	Proportion of college graduates or above	Employee
	Material input	School building area per student	Area
		Number of books per capita	Book
The output variables	Output quality	Number of kindergarten graduates	School
	Output quantity	Proportion of primary school enrolment with pre-primary education	Garden
The external variable	Economic factors	GDP per capita	City
	Demographic factors	Fertility rate	Born
	Scale factors	Average number of kindergarten students per million population	Scale
	Agglomeration factors	Number of kindergartens	Agglomeration

Regarding the selection of external variables, this paper incorporates the following dimensions: (1) Economic factors (GDP). The level of economic development affects the efficiency of rural preschool education resource allocation, and regions with higher levels of economic development pay more attention to education and sustainable development issues, resulting in higher performance of rural preschool education input. This paper selects GDP per capita as an indicator of economic development level. (2) Population factors (Born). The impact of population on educational investment has both advantages and disadvantages. This paper selects the proportion of birth population in each province and city as an indicator of population birth rate. (3) Scale factors (Scale). Kindergarten scale is closely related to the efficiency of rural preschool education resource allocation. This study selects the average number of kindergarten students per million population in each province as the measurement indicator for scale factors.(4) Agglomeration factors (Agglomeration). The agglomeration effect of kindergartens influences preschool education investment efficiency. This paper utilizes the number of kindergartens in each region as the measurement indicator for agglomeration factors.

### 3.2 Data Source

Considering the research needs and data availability, this paper utilized data from 30 provinces in China (excluding Tibet, Hong Kong, Macao, and Taiwan) from 2012 to 2020, mainly sourced from the “China Statistical Yearbook,” “China Education Statistical Yearbook,” “China Education Funds Statistical Yearbook,” and official data published by the National Bureau of Statistics. Some missing data are filled in using interpolation methods.

To distinguish between the central, eastern, and western economic regions, this paper introduces two dummy variables for differentiation. D1=1 indicates the eastern region, while D1=0 indicates the western or central region; D2=1 indicates the central region, and D2=0 indicates the eastern or western region. This paper divides the provinces involved in this study into three major regions: eastern, central, and western.

## 4 Research Method

**Fig 3:**
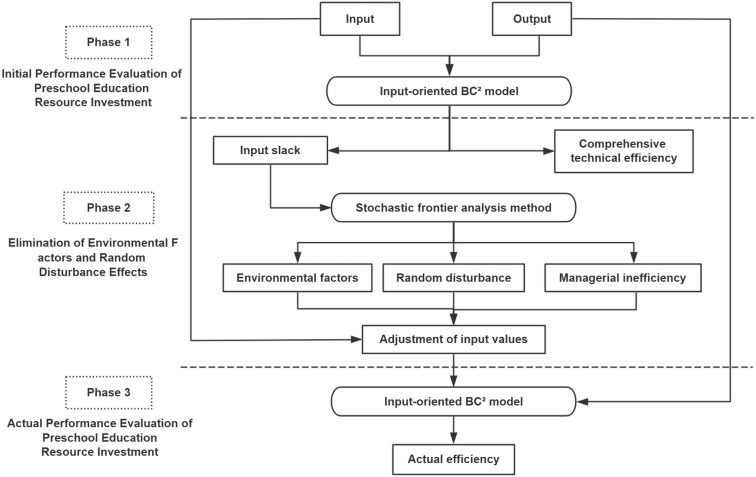
Logical framework diagram of the three-phase DEA methodology.

Data Envelopment Analysis (DEA) is a new systems analysis method developed by renowned operations researchers such as Charnes based on the concept of “relative efficiency evaluation.” It utilizes the ratio of the weighted sum of multiple output indicators to the weighted sum of multiple input indicators to reflect the effectiveness of decision-making units (DMUs) with multiple inputs and outputs. The core idea involves determining the production frontier by comparing the input-output efficiency of multiple DMUs. DMUs on the production frontier are deemed efficient, while those not on the frontier are classified as DEA-inefficient. The relative efficiency value of each DMU is then determined by measuring the distance from DEA-inefficient points to the production frontier [30]. Data Envelopment Analysis (DEA) typically includes radial models represented by CCR and BCC, as well as non-radial models represented by SBM. Addressing the limitation of traditional DEA models in handling non-discretionary factors, Fried attempted to incorporate environmental factors and random noise interference into the model, proposing the famous three-stage DEA model. As shown in Fig 3, the three-stage DEA model ameliorates the impact of external environmental factors and random errors, random disturbances, and other factors, resulting in efficiency values that are closer to reality. This research conducts static and dynamic analyses of rural preschool education resource allocation efficiency and its determinants, providing empirical evidence for improving allocation efficiency of rural preschool education resource.

### 4.1 Three-stage DEA model

#### 4.1.1 The first stage: traditional BCC model.

Considering the differences in the scale of rural preschool education input among various provinces and cities, this study adopts the BCC model, which accommodates variable returns to scale. The original input-output data were imported into DEAP 2.1 software to calculate the comprehensive efficiency, pure technical efficiency, and scale efficiency of each decision-making unit, as well as the slack values of each input indicator. Simultaneously, we focus on studying how to minimize the input while maintaining the output unchanged. For a given decision-making unit (DMU), the input-oriented BCC model can be expressed as Equation (1):


$$\min\left[\theta_{j}-\varepsilon \left(\sum_{i=1}^{m}\;s_{ij}^{-}+\sum_{r=1}^{k}\;s_{rj}^{+}\right)\right]$$

$$\text{s.t. }\left\{\begin{array}{l} \sum_{j=1}^{n}\;2mu\lambda_{j}x_{ij}=\theta_{n}x_{ij}-s_{ij}^{-},i=1,2,\cdots ,m\;\\ \sum_{j=1}^{n}\;2mu\lambda_{j}y_{rj}=y_{rj}s_{r}^{j},r=1,2,\cdots ,k\\ \sum_{j=1}^{n}2mu\lambda_{j}=1\\ \lambda_{j}≽0,s_{i}^{n-}≽0,s_{r}^{j}≽0,j=1,2,\cdots ,n \end{array}\right.\;$$ (1)

where j = 1, 2,..., n denotes the decision unit, and x and y are the input and output variables, respectively. Technical efficiency refers to the extent to which the production process of a production unit reaches the technical level of the industry. In this linear programming problem, a decision cell is efficient if θ=1, $s^{-}=s^{+}=0$; weakly efficient if θ=1, $s^{+}\neq 0$ or $s^{-}\neq 0$; and non-efficient if θ<1.

The BCC model can decompose the calculated technical efficiency (TE) into scale efficiency (SE) and pure technical efficiency (PTE), i.e., equation (2):


TE=SE×PTE (2)

#### 4.1.2 The second stage: stochastic frontier SFA model.

In the second stage, the SFA regression model is utilized to examine the effects of environmental factors, random disturbance terms, and management inefficiency. Using FRONTIER 4.1 software, modeling analysis is conducted for the slack variables of each input indicator. Slack variables represent the discrepancy between the actual production and operational inputs of each region and the inputs at the highest efficiency. These variables encapsulate the combined influences of environmental factors, random errors, and managerial inefficiencies. In the second stage, the input slack variables derived from the first stage are employed to construct the SFA model, as presented in Equation (3):


$$S_{in}=f_{i}\left(Z_{n};\beta_{i}\right)+V_{in}+U_{in}$$ (3)

$S_{in}$ represents the relaxation value of the nth input for the ith decision unit; $Z_{n}$denotes the environmental variable with $\beta_{i}$ as its coefficient. The term $V_{in}+U_{in}$ encompasses a composite error, where $V_{in}$ signifies a random disturbance term following a normal distribution $N(0,{\sigma_{\mu_{n}}}^{2})$, and $U_{in}$ represents managerial inefficiency following a normal distribution $N(0,{\sigma_{\mu_{n}}}^{2})$. In equation (3), the efficiency values include the effects of external environmental indicators $f_{i}\left(Z_{n};\beta_{i}\right)$ and the mixing error term $\;V_{in}+U_{in}$, which need to be corrected in the next step in order to exclude these effects.

Through the regression analysis of the SFA model, the effect of external environmental factors and random interference is excluded, and the revised input volume is obtained, as shown in equation (4):


$$X_{in}^{A}=X_{in}+\left[max(f(Z_{i};{\hat{\beta }}_{n}))-f(Z_{i};{\hat{\beta }}_{n})\right]+\left[max(\nu_{ni})-\nu_{ni}\right]$$ (4)

Where $X_{in}$ represents the actual input value; $X_{in}^{A}$ represents the adjusted input value; $\left[max(\nu_{ni})-\nu_{ni}\right]$ means that all decision units are at the same level. $\left[max(f(Z_{i};{\hat{\beta }}_{n}))-f(Z_{i};{\hat{\beta }}_{n})\right]$ is an adjustment for environmental factors.

#### 4.1.3 The third stage: the model is replaced again after adjustment.

When evaluating the efficiency of decision-making units (DMUs), by controlling for the effects of environmental and random factors and employing adjusted input-output variables, the resulting efficiency estimates offer a more objective and precise reflection of the true efficiency level compared to unadjusted data.

### 4.2 Three-stage malmquist model

The DEA-BCC model is limited to static analysis. To achieve a comprehensive analysis of rural preschool education resource allocation efficiency, Malmquist index analysis should also be incorporated. The Malmquist index measures the dynamic efficiency of a system with a multi-input and multi-output structure, capturing the relative efficiency of decision-making units (DMUs) over two periods. Specifically, it indicates whether efficiency has improved, remained unchanged, or deteriorated in the subsequent period compared to the previous one.

The Malmquist Index model addresses the limitation of traditional DEA models, which are confined to static efficiency analysis, offering a dynamic perspective on efficiency information. It has been widely applied in various fields for efficiency measurement. After completing the three-stage DEA static analysis of rural preschool education allocation efficiency, this paper develops a three-stage Malmquist framework to dynamically investigate temporal variations in allocation efficiency. The objective is to systematically examine how rural preschool education resources allocation efficiency evolves across different periods.

Methodologically, the basic approach of this paper is similar to the previous three-stage DEA model. In the first stage, the traditional Malmquist model is used to analyze changes in total factor productivity. In the second stage, a stochastic frontier SFA model is employed for estimation. In the third stage, the adjusted input variables and original output variables are fed back into the Malmquist model for calculation.

The Malmquist Index model represents a widely adopted approach for assessing dynamic changes in educational resource allocation, decomposed into two primary components: efficiency change (Effch) and technological change (Techch). Efficiency change (Effch) can be further decomposed into pure technical efficiency change (PEch) and scale efficiency change (SEch), as delineated in Equation (5):


TFPch = Effch × TEch = PEch × SEch × TEch (5)

Wherein, TFPch (Total Factor Productivity change) denotes the overall shift in production efficiency represents the change in production efficiency. When TFPch is greater than 1, it indicates that production efficiency has improved; otherwise, it indicates a decline in production efficiency. Effch (Efficiency change) quantifies the degree to which a decision-making unit (DMU) optimizes its utilization of existing technology. An Effch value exceeding 1 signifies an enhancement in technical efficiency, while a value below 1 suggests a deterioration. Tech (Technological change) captures the impact of production frontier movements on productivity growth. A Tech value above 1 reflects technological advancement, whereas a value below 1 denotes technological regression. PEch (Pure Technical Efficiency change) measures the efficiency gains arising from managerial and allocative improvements. A PEch value greater than 1 signals enhanced technology application efficiency, while a value less than 1 implies reduced efficiency.SEch (Scale Efficiency change) reflects the influence of economies of scale on productivity. An SEch value above 1 signifies scale optimization, whereas a value below 1 indicates scale diseconomies.

Collectively, if Effch, Techch, PEch, and SEch all exceed 1, they collectively drive total factor productivity growth; conversely, values below 1 suggest no such growth.

## 5 Empirical analysis

### 5.1 Static analysis of three-stage DEA model

#### 5.1.1 The first stage: traditional DEA model.

Using DEAP2.1 software, the performance of rural preschool education input in China’s 30 provinces (excluding Tibet, Hong Kong, Macao, and Taiwan) from 2012 to 2020 was measured, as shown in Table 2. The efficiency levels vary significantly among the eastern, central, and western regions. From the national average value of comprehensive technical efficiency from 2012 to 2020, it can be found that the impact of reform measures on rural preschool education has been limited. The main difficulties in the supply of rural preschool education resources in China stem from the defect of the system and the imperfection of policies, which are specifically manifested as follows: First, in recent years, the state has further adjusted the school system and layout of primary and secondary schools, merging existing rural primary schools and canceling preschool classes attached to village primary schools. These reform measures have triggered a series of negative reactions, exacerbating the problem of rural preschool education resource supply; second, the supply of preschool education shows an urban-oriented tendency, vigorously developing urban preschool education. By 2020, a supply structure with “public kindergartens as demonstration and private as the main body” has basically emerged, but this structure is not suitable for the vast rural areas, and the contradiction between supply and demand in rural preschool education remains severe.

**Table 2:** rural Preschool Education Resources Allocation Efficiency in China.

Region	Province	The first stage			The third stage		
		TE	PTE	SE	TE	PTE	SE
Eastern China	Beijing	0.438	0.59	0.743	0.818	0.906	0.904
	Tianjin	0.528	0.763	0.697	0.852	0.97	0.878
	Hebei	0.584	0.74	0.792	0.91	0.975	0.933
	Liaoning	0.567	0.729	0.777	0.855	0.917	0.932
	Shanghai	0.425	0.568	0.752	0.783	0.859	0.913
	Jiangsu	0.474	0.619	0.766	0.828	0.912	0.909
	Zhejiang	0.524	0.703	0.743	0.775	0.862	0.899
	Fujian	0.712	0.836	0.846	0.918	0.965	0.95
	Shandong	0.547	0.744	0.732	0.87	0.984	0.883
	Guangdong	0.66	0.732	0.895	0.936	0.972	0.963
	Hainan	0.613	0.689	0.895	0.791	0.84	0.944
	Mean	0.552	0.701	0.785	0.849	0.924	0.919
Central China	Shanxi	0.56	0.679	0.825	0.881	0.897	0.981
	Jilin	0.531	0.652	0.822	0.761	0.798	0.954
	Heilongjiang	0.504	0.624	0.816	0.764	0.808	0.945
	Anhui	0.617	0.719	0.847	0.904	0.954	0.946
	Jiangxi	0.706	0.789	0.891	0.897	0.92	0.974
	Henan	0.762	0.882	0.861	0.908	0.969	0.937
	Hubei	0.771	0.901	0.858	0.933	0.975	0.957
	Hunan	0.715	0.954	0.749	0.903	0.993	0.909
	Mean	0.646	0.775	0.834	0.869	0.914	0.95
Western China	Inner Mongolia	0.486	0.647	0.762	0.722	0.789	0.915
	Chongqing	0.727	0.818	0.881	0.901	0.936	0.962
	Sichuan	0.636	0.686	0.93	0.804	0.847	0.952
	Guangxi	0.773	0.835	0.922	0.923	0.955	0.966
	Guizhou	0.58	0.614	0.941	0.785	0.832	0.949
	Yunnan	0.589	0.625	0.944	0.754	0.821	0.924
	Shanxi	0.552	0.676	0.814	0.732	0.78	0.938
	Gansu	0.373	0.447	0.85	0.647	0.741	0.883
	Qinghai	0.549	0.583	0.95	0.717	0.761	0.946
	Ningxia	0.561	0.642	0.877	0.852	0.905	0.944
	Xinjiang	0.629	0.758	0.846	0.88	0.936	0.94
	Mean	0.587	0.667	0.883	0.793	0.846	0.938
National average		0.590	0.708	0.834	0.834	0.893	0.934

Note: TE=PTE×SE; S.1 is the efficiency value of stage I, and S.3 is the efficiency value of stage III

As indicated in Table 2, overall, from 2012 to 2020, the national averages for comprehensive technical efficiency, pure technical efficiency, and scale efficiency in the first phase were 0.590, 0.708, and 0.834, respectively. Among them, scale efficiency is superior to pure technical efficiency, indicating that pure technical efficiency is a significant factor contributing to the low performance of preschool education resource investment in China. From an inter-provincial perspective, there are also significant differences in the degree of improvement in preschool education allocation efficiency among different provinces and cities. Preschool education allocation efficiency in eastern provinces and cities has generally improved, with comprehensive technical efficiency increasing from 0.552 to 0.849, while the improvement in central and western provinces and cities is not significant, with comprehensive technical efficiency only increasing from 0.646 and 0.587 to 0.869 and 0.793, respectively. However, the results do not strip away environmental and random factors, and the situation of rural preschool education allocation efficiency in each province and city is not reflected accurately enough. Future research should focus on eliminating the interference of external environmental factors to provide a more precise assessment of the allocation efficiency of rural preschool education.

#### 5.1.2 The second stage: SFA model analysis.

The explained variables are the redundancy values of each input variable in the first-stage DEA model, and the explanatory variables are the environmental variables. The SFA regression is conducted using Frontier4.1 software, and the regression results are shown in Table 3. The likelihood ratio test (LR test) in the SFA regression results indicates that the estimation model fits the data well. Most of the coefficients of the environmental variables on the input variables are statistically significant, indicating that external environmental variables significantly impact the redundancy of each input variable. The γ value of each preschool education input slack variable is as high as 0.93, indicating that management factors play a dominant role in influencing input redundancy, Therefore, it is essential to control for environmental and random factors to more accurately assess the underlying performance.

**Table 3:** Second stage: SFA regression results.

	Average education expenditure	Proportion of junior college graduates or above	School building area per student	Number of books per capita
Constant	-11058.949***	-40.045***	-6.520***	-6.520***
Economic development level	-148.749***	-0.572***	-0.084***	-0.031***
Fertility rate	328.418***	1.678***	0.292***	0.080*
Number of kindergartens	22.092***	0.059***	0.007**	0.014***
Average number of kindergarten students per million population	-6.532***	-0.057***	-0.007***	-0.0027
σ²	45677218.000	563.720	13.480	45.185
γ	0.947	0.961	0.937	0.986
LR unilateral check value	404.941***	251.485***	171.660***	446.044***

Note. ***, **, and*shows significance at the 1% level, 5% level, and 10% level, respectively

From Table 3, it can be known that the input redundancy is regarded as the opportunity cost of rural preschool education resource allocation in each province, that is, when the regression coefficient is positive, it will lead to either waste of inputs or a reduction in potential output, thereby hindering the improvement of the efficiency of rural preschool education resource allocation. Conversely, a negative regression coefficient suggests that the input redundancy is associated with enhanced efficiency in the allocation of rural preschool education resources.The specific impact of environmental factors is as follows:

The regression results show that the economic development level (GDP) exhibits negative coefficients for the slack variables of per-student education expenditure, the proportion of teachers graduated from junior college or above, per-student school building area, and per-student books (volumes), and all are significant at the 1% level, indicating that this variable has a positive impact on these four inputs. This also verifies the conclusion of Wang Yongjie’s research [31].

The birth rate (born) exhibits positive coefficients for the slack variables of per-student education expenditure, the proportion of teachers graduated from junior college or above, per-student school building area, and per-student books (volumes), indicating that an increase in the birth rate has a negative impact on these four inputs. This is consistent with the findings of Jun’e Liu [32].

The number of kindergartens (agglomeration) has positive coefficients for the slack variables of per-student education expenditure, the proportion of teachers graduated from junior college or above, per-student school building area, and per-student books (volumes), and is significant at the 1% level and passes the significance test, which is implying a detrimental effect on resource allocation efficiency. This is consistent with the empirical conclusion of Greier [33].

The regression coefficients of the average number of kindergarten students per million population (scale) on the slack variables of input indicators such as per-student education expenditure, the proportion of teachers graduated from junior college or above, and per-student school building area are negative and are statistically significant at the t-test level. A higher average number of kindergarten students per million population more effectively reduces redundancy in preschool education inputs and mitigates input factor waste. This is consistent with the research findings of Rajchanovska [34].

#### 5.1.3 The third stage: adjusted DEA empirical results.

After processing the data obtained from the second stage, the input values after excluding environmental variables and random factors are obtained. Using the new input values and the original output values, the adjusted level of rural preschool education resource allocation efficiency for each province, region, and city through DEAP2.1 software is shown in Table 3.

According to the adjusted data, the performance of provinces and municipalities in terms of preschool education resource input has improved significantly. The mean value of comprehensive technical efficiency increased from 0.590 in stage one to 0.834, the mean value of pure technical efficiency increased from 0.708 to 0.893, and the mean value of scale efficiency grew from 0.834 to 0.934.This indicates that the performance of preschool education resource inputs in each province, region, and city was improved to a certain extent after the environmental factors were eliminated, and this improvement was statistically significant. It is also worth noting that in the adjusted data, scale efficiency is higher than pure technical efficiency. This suggests that deficiencies in pure technical efficiency directly constrain the improvement of comprehensive efficiency. The significant difference in the adjustment of preschool education resource allocation efficiency between provinces and cities is mainly due to the fact that the provinces and cities have their own stochastic factors that are different and have an impact on the preschool education resource allocation efficiency, which suggests that the third stage of the measurement is more reasonable in reflecting the actual level of management efficiency.

**Fig 4:**
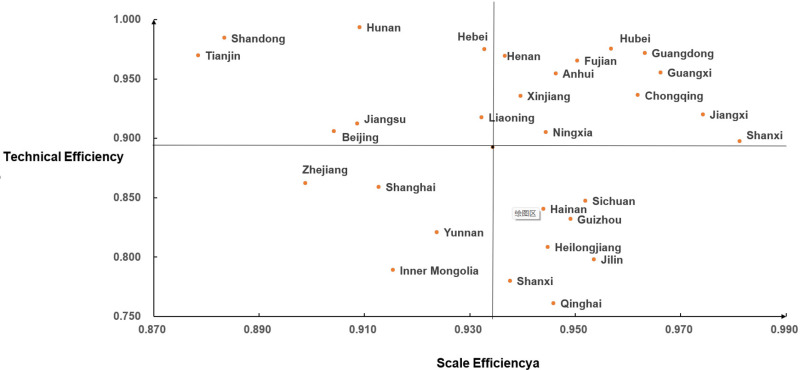
Distribution of pure technical efficiency and scale efficiency of preschool education resource allocation.

To further explore the strategy of improving the performance of rural preschool education investment, this paper utilizes the average values of pure technical efficiency and scale efficiency measured in three stages in Table 2 as the critical point, and the performance of rural preschool education investment in each province and city can be categorized into four types, as shown in Fig 4 (regions with comprehensive technical efficiency on the production frontier are not shown in the figure). Type One, designated as the ‘Double High Type,’ is characterized by both pure technical efficiency and scale efficiency exceeding the national average, including 11 provinces and cities, such as Shanxi, Jiangxi, Henan, and Guangxi, accounting for 36.7% of all provinces, regions, and cities. Among these areas, the performance of preschool education investment is relatively effective. Type Two is called “High-Low Type,” with pure technical efficiency higher than the national average but scale efficiency lower than the national average, seven provinces included: Beijing, Jiangsu, Liaoning, Shandong, Hunan, Tianjin, and Hebei. The direction for improving efficiency in these areas is to promote the centralized allocation of resources and optimize the input-output ratio of rural preschool education resources. Type Three is called “Low-High Type,” with scale efficiency higher than the national average but pure technical efficiency lower than the national average, represented by cities such as Sichuan, Shaanxi, Guizhou, Heilongjiang, Jilin, Hainan, and Qinghai. For these areas, priority should be given to optimizing resource allocation, enhancing production management practices, and fostering technological innovation. Type Four, termed the ‘Double Low Type,’ is characterized by both scale efficiency and pure technical efficiency falling below the national average, exemplified by Yunnan, Inner Mongolia, Shanghai, Zhejiang, and Gansu. It is more arduous to promote the efficiency of rural preschool education resource allocation in these areas in the future, calling for improvements in resource allocation capabilities through expanding scale and strengthening management.

### 5.2 Dynamic analysis of three-stage malmquist inde

To further empirically examine the longitudinal dynamics of decision-making unit efficiency and investigate the underlying drivers of variations in rural preschool education investment performance, this study employs panel data on rural preschool education resource allocation across 30 provinces and municipalities in China from 2012 to 2020. Using DEA-SOLVERPRO 5.0 software to calculate the Malmquist index, the changes in the performance of rural preschool education investment in various regions are obtained, as shown in Table 4.

**Table 4:** Malmquist index and its decomposition of preschool education resource allocation in China.

Region	Province	The first stage			The third stage		
		M Productivity index	TC index	TEC index	M Productivity index	TC index	TEC index
Eastern China	Beijing	1.011	0.952	1.064	1.001	0.987	1.014
	Tianjin	0.953	0.952	1.002	0.975	0.989	0.986
	Hebei	0.968	0.933	1.047	0.986	0.986	1
	Liaoning	0.958	0.932	1.029	0.982	0.977	1.005
	Shanghai	1.012	0.951	1.067	1.011	0.989	1.023
	Jiangsu	0.955	0.949	1.006	0.97	0.976	0.994
	Zhejiang	0.948	0.952	0.997	0.978	0.988	0.99
	Fujian	0.945	0.95	0.997	0.982	0.98	1.002
	Shandong	0.942	0.943	0.999	0.982	0.984	0.998
	Guangdong	0.935	0.949	0.987	0.993	0.993	1
	Hainan	0.963	0.954	1.008	0.98	0.986	0.993
	Mean	0.963	0.947	1.018	0.985	0.985	1.001
Central China	Shanxi	0.951	0.956	1.011	0.972	0.976	0.996
	Jilin	0.961	0.953	1.01	0.967	0.987	0.98
	Heilongjiang	0.957	0.947	1.012	0.965	0.979	0.985
	Anhui	0.914	0.934	0.984	0.974	0.982	0.993
	Jiangxi	0.931	0.951	0.977	0.979	0.983	0.996
	Henan	0.943	0.943	1	0.979	0.979	1
	Hubei	0.981	0.963	1.02	0.998	0.992	1.007
	Hunan	0.971	0.955	1.02	0.987	0.989	0.999
	Mean	0.951	0.95	1.004	0.978	0.983	0.994
Western China	Inner Mongolia	0.948	0.954	1	0.966	0.988	0.978
	Chongqing	0.934	0.952	0.981	0.977	0.985	0.992
	Sichuan	0.936	0.935	1.002	0.999	0.982	1.017
	Guangxi	0.954	0.954	1	0.981	0.981	1
	Guizhou	0.924	0.955	0.971	0.989	0.982	1.006
	Yunnan	0.919	0.945	0.971	0.992	0.984	1.007
	Shanxi	0.953	0.952	1.001	0.974	0.987	0.987
	Gansu	1.009	0.903	1.119	1.029	0.982	1.048
	Qinghai	0.964	0.951	1.024	0.998	0.976	1.023
	Ningxia	0.928	0.954	0.981	0.993	0.976	1.017
	Xinjiang	0.974	0.94	1.05	0.991	0.968	1.024
	Mean	0.949	0.945	1.009	0.99	0.981	1.009
National average		0.955	0.947	1.011	0.985	0.983	1.002

Nationwide, during the third stage (2012-2020), the overall productivity, technological progress, and changes in technical efficiency of China’s rural preschool education resource allocation were all superior to the first phase. After ignoring environmental factors and random factors, the overall authenticity of the efficiency of rural preschool resource allocation is enhanced, highlighting the efficacy of management decisions. The adjusted Malmquist index exhibits an average value of 0.985, corresponding to an average annual growth rate of 3.1%, with an average growth rate of 3.1%. On the structural level, the efficiency of technological progress change and the change in technical efficiency after adjustment are 0.947 and 0.983 respectively, indicating that technological improvement is the core driving factor in improving the productivity of rural preschool education resource allocation, while technical efficiency is the main reason restricting total factor productivity.

On the provincial level, only three provinces are considered to have effective rural preschool education resource allocation efficiency in the first phase. After stripping away environmental and random factors, the Total Factor Productivity Change Index (TFPch) of all provinces across the country has gradually increased, mainly owing to the limited fiscal capacity of western regional governments. However, some regions (e.g., Inner Mongolia, Shaanxi, Chongqing, Gansu, Qinghai, etc.) have shown a downward trend in technical efficiency, with an average technical efficiency value of less than 1. It is worth noting that the efficiency of technological progress in most provinces across the country has improved in the third phase.The possible reasons lie in the following: The rural preschool education sector in China is primarily under the responsibility of local governments, but due to varying levels of economic development across provinces, there are differences in their capacity to invest in education. This results in disparities in the initial levels of educational investment. Eastern provinces with higher economic development levels have the ability to continuously increase investment, reaching preschool education standardization requirements at an earlier stage. For these regions, further investments would only lead to waste. In contrast, central and western provinces face inefficiencies due to insufficient investment, which constrains the improvement of educational resource allocation efficiency.

On the regional level, the Malmquist index for the efficiency of rural preschool education resource investment in China has demonstrated notable improvement, ranking from high to low as eastern, central, and western regions. Among them, the role of technological progress in promoting total factor productivity improvement is most evident in the eastern region. It benefits from a higher level of economic development in management innovation and technological progress, and the introduction of advanced equipment and talent in rural preschool education resource allocation; The continuous deterioration of scale efficiency in the western region has led to the largest annual decline in comprehensive technical efficiency. This may be due to the weakness in government’s financial strength in the western region is weak, and the public finance budget is mainly used to maintain regular expenditures, basically belonging to the category of “ mouth-feeding budget.” Indeed, with a low level of marketization and low participation of social capital, investment funds for rural preschool education have been severely insufficient for many years, so there is little impetus for improving scale efficiency.

## 6 Conclusion and recommendation

### 6.1 Conclusions

Based on cross-sectional and panel data from 2012 to 2020 in China, this paper employ a three-stage DEA model and the Malmquist index to measure and analyze the trends in comprehensive technical efficiency and total factor productivity of rural preschool education investment performance in 30 provinces, regions, and cities across the country from both static and dynamic dimensions, after eliminating the influence of environmental and random factors. The following conclusions are drawn:

Firstly, environmental and random disturbance factors have a significant impact on the performance of rural preschool education investment. Through the SFA regression analysis in Stage Two, economic factors and scale factors will reduce input slack values, which is beneficial to improving the performance of rural preschool education resource investment. In contrast, increases in population factors and agglomeration factors lead to an increase in input slack values, reducing the performance of rural preschool education resource investment.

Secondly, the performance of rural preschool education resource investment in China has been improved. After eliminating the influence of environmental and random factors, the average performance of rural preschool education investment increased from 0.590 to 0.893, indicating that there has been an improvement in performance. Among them, deficiencies in pure technical efficiency serve as the primary constraint on comprehensive efficiency improvement.

Thirdly, a classification framework was adopted, using the mean values of pure technical efficiency and scale efficiency as critical thresholds, categorizing the 30 provinces and cities into four efficiency types: “Double High,” “High-Low,” “Low-High,” and “Double Low.” Approximately one-third (36.7%) of provinces and regions exhibit a ‘Double High’ efficiency configuration.

Fourthly, technological progress is the main driving force behind the growth of total factor productivity. From 2012 to 2020, the annual average growth rate of total factor productivity (TFP) in rural preschool education investment reached 3.1%. The contribution of technological progress to the growth of total factor productivity is significantly higher than that of comprehensive technical efficiency during the same period.

Fifthly, there are significant regional differences in the performance of rural preschool education investment. After excluding the effect of environmental factors, the average comprehensive efficiency of the eastern region in China is the highest, followed by the central and western regions. There are obvious regional disparities in the performance of rural preschool education investment, especially the rural preschool education resource investment performance of provinces and cities along the southeast coast is significantly higher than the national level.

### 6.2 Suggestions

From an economic policy standpoint, strengthening the financial security mechanism for rural preschool education is imperative.To begin with, it is crucial to enhance the financial input of central and provincial governments in rural preschool education and utilize the special funds with the support from the central financial support for the development of rural preschool education. Furthermore, the government should introduce national standards for per-student public funds in rural preschool education at the national level, and encourage regional governments to raise and implement standards for per-student financial allocations, per-student public funds, and per-student subsidy standards. Next, establish and improve regulatory institutions for the allocation of educational resources.Central and provincial governments should strengthen financial investment, thus, optimizing the subsidy system is essential. According to the policy requirements of the poverty alleviation transition period, economically disadvantaged children in subsidized kindergartens should receive welfare support, including tuition and living cost waivers. What’s more, continue to implement the nutrition improvement plan for rural preschool children, and local governments can further expand the implementation scope according to actual conditions. Provinces should provide fiscal support to regions facing affordability challenges in preschool education expenditure. Lastly, implement an adjustment mechanism for rural preschool education funding. The government should focus on supporting kindergartens at the grassroots village level in remote mountainous areas and ethnic regions, while improving the funding security mechanism that combines hardware construction such as equipment updating and software construction such as the resource of teachers and curriculum, so to attract students to return to rural areas. The Investment and allocation of resources targeted should suit the different situations of each province and city respectively. Local governments should reasonably allocate a set of resources to various regions based on local actual conditions, address current shortages in educational resources, and optimize resource allocation in regions with educational resource redundancies.

From the perspective of scale factors, we should accelerate the construction of the preschool education teacher workforce. First, government-affiliated institutions should allocate more staffing quotas to rural preschools. Based on the changes in the number of children in kindergartens in each province (city), rural kindergarten staffing should be adjusted internally within the education system first, giving priority to those from other systems such as health and culture; surplus teacher staffing from the rural compulsory education stage should be given priority to rural kindergartens; furthermore, explore setting up a separate kindergarten teacher staffing category to increase the proportion of rural kindergarten teacher staffing. Next, gradually improve the treatment of rural kindergarten teachers. Appropriately increase the proportion of senior professional titles for rural kindergarten teachers; establish a kindergarten teacher honor system, and give spiritual and material rewards to rural kindergarten principals and full-time teachers with outstanding contributions according to relevant regulations. Indeed, rural kindergarten teachers should be ensured to enjoy the same treatment as rural compulsory education teachers. The wage level of private kindergarten teachers should be determined referring to the wage level of rural public kindergarten teachers to achieve the target of equal pay for equal work between private and public kindergarten teachers, and between staff and non-staff teachers. Lastly, enrich the channels for supplementing rural preschool teacher resources. Qualified rural preschool teachers can be supplemented through the implementation of targeted training plans for publicly funded normal students, publicly funded male preschool teacher training plans, and retraining for surplus teachers in rural primary and secondary schools. In targeted training and open recruitment, focus on attracting local graduates to effectively supplement and stabilize the preschool teacher workforce.

From the perspective of agglomeration factors, we should rationally plan the supply of rural preschool education places. To begin with, carry out timely planning and reservation of land for rural kindergartens. Considering the impact of China’s new urbanization process and the Rural Revitalization Strategy, combined with the implementation of the fertility policy and local realities, revise and adjust the standards for matching educational places for residential community populations in a timely manner; prioritize the conversion of idle school buildings in primary and secondary schools into kindergartens, and attach kindergartens to rural primary schools. Next, scientifically plan and expand the total supply of kindergarten places. The number of rural kindergartens through multiple channels and the proportion of public kindergartens should be increased year by year, implemented by expand in group operations for public kindergartens, public educational resources. Two models are used to achieve group operations for public kindergartens: One is to set high-quality public kindergartens as the group’s main campus, with newly built kindergartens or poorly developed kindergartens with independent legal person status as branches, and promote the development of branches by dispatching principals and teachers from the main campus; the other is to use high-quality public kindergartens as the group’s main campus, with the principal of the main campus serving as the legal person of the newly built kindergartens. The group operation model under the same legal person is conducive to more flexible and efficient mobilization of resources throughout the group, fully leveraging its radiating and driving role. Staffing should be strategically leveraged to prioritize key positions (e.g., branch principals, health officers, financial staff, and core teachers), constructing a talent network for group operations and maximizing the institutional advantages of public kindergartens. Lastly, reasonably arrange resources for kindergartens in remote rural areas. Strengthen the construction in aspects such as teacher access, qualifications for operating kindergartens, kindergarten construction, and financial investment for rural kindergartens, to promote the standardization of preschool education resource allocation in rural areas both in macro planning and internal management.

From the perspective of demographic factors, dynamically monitor changes in the preschool education eligible population. To start with, establish an inter-cycle regulatory coordinating body for urban and rural preschool education resources. Strengthen comprehensive planning for the balanced and integrated development of urban and rural preschool education by ccoordinating the roles and responsibilities of the Organization Establishment Committee, Ministry of Human Resources and Social Security, the Ministry of Industry and Information Technology, the Ministry of Education, the National Development and Reform Commission, the Ministry of Finance, the Ministry of Housing and Urban-Rural Development, and social organizations, further optimizing teacher staffing management, school funding input, daily supervision services, etc. Next, refine household registration, archival, and other management services, and carry out urban and rural preschool education resource layout and allocation fairly, efficiently, and with quality. Indeed, establish and update dynamically a basic database of the rural preschool population. Regular monitoring requires comprehensive collection and organization of information on fertility rates and population movements in various regions, precisely and dynamically monitor changes in the number of school-age children and students in compulsory education. The dominant monitoring should focus on should focus on the impact of important changes such as adjustments to fertility policies on future changes in the number of school-age children and students in preschool education. Lastly, fairly, efficiently, and effectively allocate urban and rural preschool education resources. On one hand, government departments and relevant institutions monitor population movements, manage and utilize preschool education resources more efficiently, adhering to dynamic regulation to achieve supply-demand adaptation. On the other hand, public disclosure of rural preschool education resource supply-demand information facilitates informed educational choices by the public.

### 6.3 Outlook

This study utilizes an input-oriented three-stage DEA model and the Malmquist index to conduct static and dynamic analysis of the performance of rural preschool education resource inputs in 30 provinces in China (excluding Tibet, Hong Kong, Macao, and Taiwan). Given the limitations of research capacity and time constraints, future research needs to be improved in the following aspects. Firstly, beyond data envelopment analysis (DEA) for empirical research, future studies could incorporate theories and methodologies from interdisciplinary perspectives to enrich research perspectives and means. For example, sociological survey methods can be used to deeply analyze the fairness of educational resource allocation, or the economic cost-benefit analysis to evaluate the effects of educational investment, and geospatial analysis methods to study the spatial layout of educational resources. Secondly, this study focuses on regional equalization disparities but does not address urban-rural equity measurements, which is also worth further research. Lastly, due to the unavailability of data, some indicators have not been included in the evaluation index system, such as indicators related to the innovation of education and teaching and the personalization of student development. Therefore, future research should pay attention to these aspects.
